# A surgical case of eosinophilic angiocentric fibrosis of the lung

**DOI:** 10.1186/s40792-015-0055-z

**Published:** 2015-06-17

**Authors:** Keigo Okamoto, Makoto Motoishi, Ryosuke Kaku, Satoru Sawai, Jun Hanaoka

**Affiliations:** Department of General Thoracic Surgery, National Hospital Organization Kyoto Medical Center, 1-1 Mukaihatacho Fukakusa, Fushimi, Kyoto, 612-0862 Japan; Department of General Thoracic Surgery, Shiga University of Medical Science, Tsukinowacho Seta Otsu, Shiga, 520-2121 Japan

**Keywords:** Eosinophilic angiocentric fibrosis, Lung cancer, Chronic cough, Hilar region mass

## Abstract

Eosinophilic angiocentric fibrosis (EAF) is an uncommon inflammatory disease that develops from the respiratory organs and affects them. Almost all reports about EAF describe lesions affecting the upper respiratory tract. We present the first case of EAF of the lung treated by surgical excision. A 69-year-old female consulted our hospital following the detection of an abnormal chest shadow with chronic cough. Chest computed tomography showed a pulmonary growing mass in the right hilar area, which corresponded to an enhanced accumulation on positron emission tomography. We doubted a pulmonary malignant tumor and performed a right upper lobectomy. Pathological and other clinical presentations revealed EAF of the lung without coexisting systemic diseases. The patient had an uncomplicated postoperative course, and the presenting cough had improved. EAF can involve the lung and cause symptomatic airway obstruction. For a hilar region mass with imaging characteristics similar to those of lung cancer, a differential diagnosis must be considered.

## Background

Eosinophilic angiocentric fibrosis (EAF) is a rare disorder typically involving the upper respiratory tract, although it may very rarely affect the lower respiratory tract. Furthermore, EAF lacks an established treatment. Almost all reported cases were surgically treated and pulmonary lesions have never been reported. EAF is considered a progressive benign disorder, and the diagnosis is chiefly based on histopathological findings [1] more specifically the presence of an inflammatory infiltrate with numerous eosinophils and fibro-inflammatory lesions. The perivascular fibrotic changes are characterized by a whorled, “onion skin”-type pattern [1, 2].

The etiology of this progressive disorder is unknown [3]. It has been suggested that several conditions act as predisposing factors, but the evidence in this regard is lacking. We present the first case of EAF manifesting as a pulmonary lesion.

## Case presentation

**Fig. 1:**
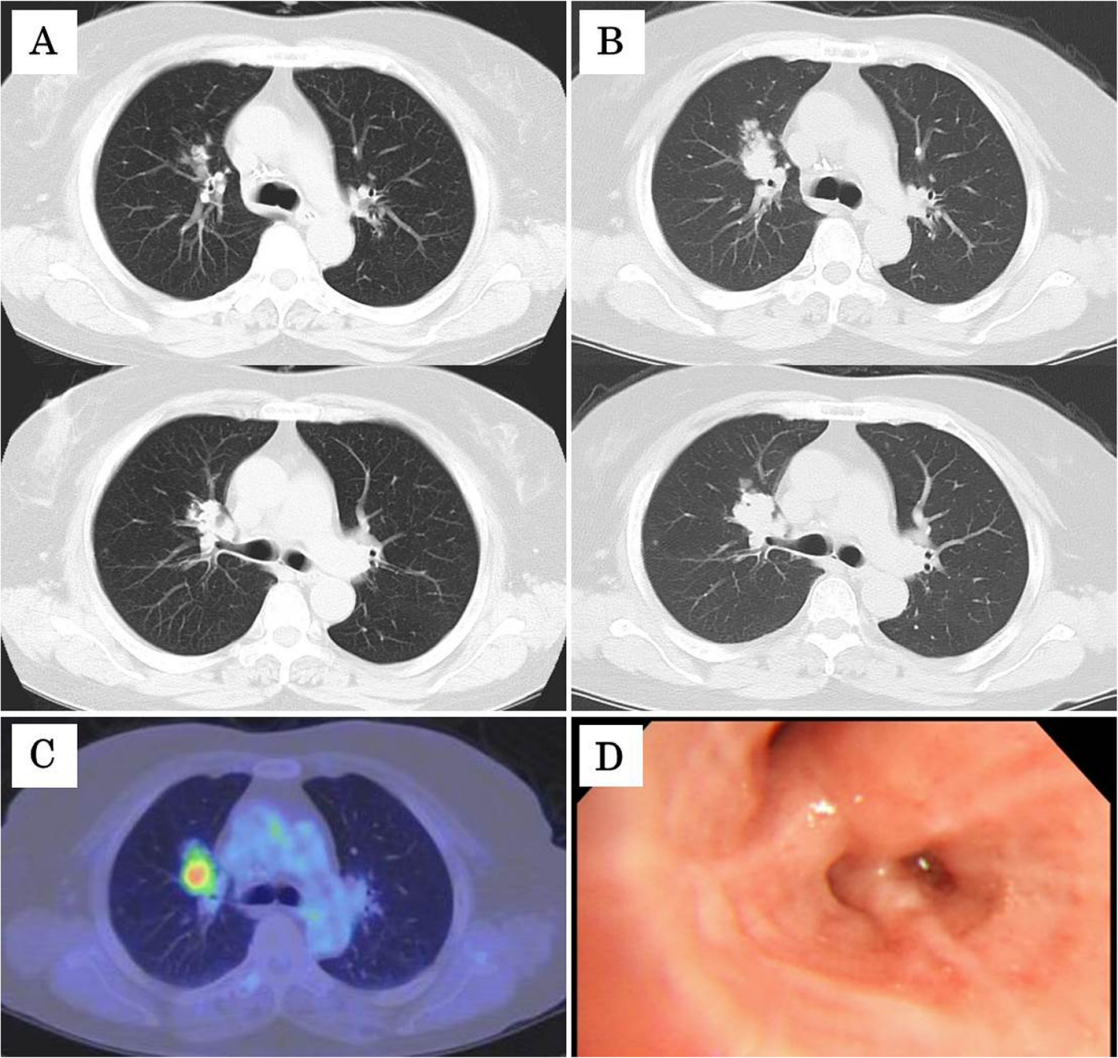
**a** Original CT scan obtained 3 years ago showed a pulmonary nodule in the upper area of the right lung. **b** CT scan on admission showed an enlarged mass. **c** FDG-PET indicated high accumulation in the mass region (SUV max = 6.5). **d** Bronchoscopic examination revealed narrowing of the superior bronchial ostia. The bronchial mucosa was macroscopically normal

A 69-year-old woman consulted our hospital following the detection of an abnormal chest shadow. She had a history of chronic cough despite having never smoked. She did not have a history of trauma or prior thoracic surgery. Laboratory examinations did not reveal abnormalities that would have indicated organ disorder or active inflammatory processes. Using chest computed tomography (CT), we identified a localized pulmonary mass with a diameter of 30 mm in the right lung hilar region. Retrospectively, the mass was visibly larger compared with that on a CT image obtained 3 years earlier (Fig. 1a, b). On ^18^F-fluoro-deoxy-glucose positron emission tomography (FDG-PET), the mass showed an enhanced accumulation of contrast (Fig. 1c). To establish a tissue diagnosis, we performed a bronchoscopic examination. Slight narrowing of the superior bronchial ostia by extrinsic displacement was observed (Fig. 1d). Specimens obtained by bronchial mucosal and transbronchial lung biopsy indicated only normal tissue. However, we were concerned about the possibility of primary lung cancer and performed video-assisted thoracic surgery with right upper lobectomy. The tumor surface was well circumscribed and farm ash-gray. There was no gross invasion into the pulmonary artery and superior bronchus. Using intraoperative frozen section, we determined that this localized pulmonary tumor was benign. Having resected the superior bronchus, we pathologically confirmed that the bronchial mucosa was normal. The patient had an uncomplicated postoperative course and was discharged 1 week later.

**Fig. 2:**
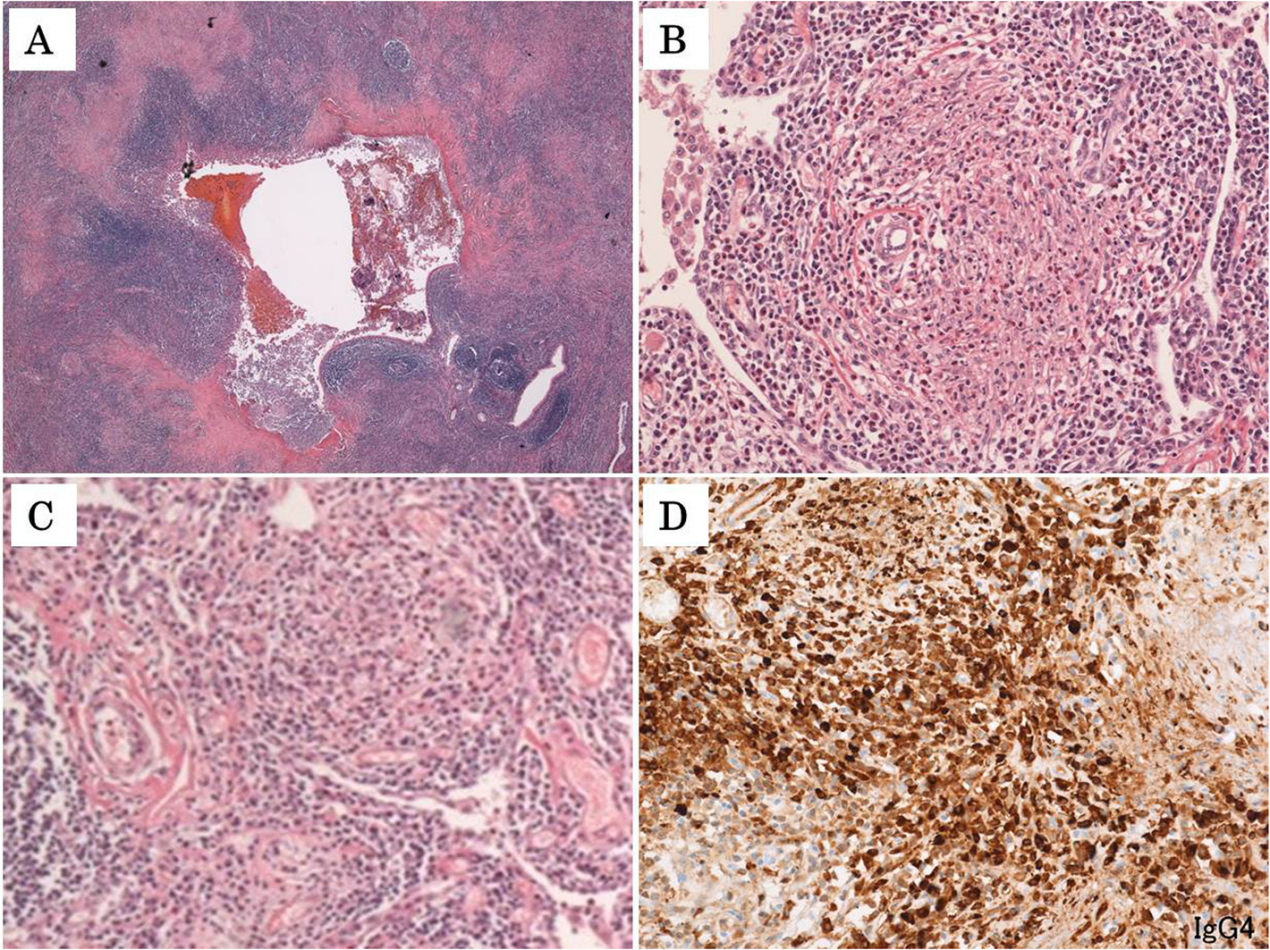
Pathological analysis showed **a** a dense bronchocentric inflammatory infiltrate with numerous inflammatory cells and perivascular fibrosis. **b** Fibro-inflammatory lesion (perivascular fibrosis) surrounding the vessel in a whorled pattern. Numerous eosinophils infiltrate the lesion. **c** A fibrotic nodule with the “onion-skin” fibrosis pattern that is pathognomonic for eosinophilic angiocentric fibrosis. **d** Immunohistochemical staining for IgG4 revealed a large number of IgG4-positive cells (400 cells per high-power field)

Histopathological analysis revealed a dense inflammatory infiltrate with numerous eosinophils and perivascular fibrosis but without signs of necrotizing vasculitis or giant cell formation. The fibro-inflammatory lesions displayed a characteristic whorled, onion skin-like fibrotic pattern, which is a specific finding in EAF. Immunostaining showed a large amount of cells (400 cells per high-power field) that were positive for immunoglobulin G4 (IgG4) (Fig. 2).

We suspected the concurrent presence of a systemic disease, such as IgG4-related disorders and granulomatosis with polyangitis (GPA), and repeated blood tests using preoperative preserved blood. Serological assays for IgG, IgE, IgG4, cytoplasmic anti-neutrophil antibody (c-ANCA), as well as urinalysis, were all negative. The only positive finding was the presence of perinuclear anti-neutrophil antibodies (p-ANCA, 19.1 U/mL). However, there were no other clinical findings, such as retroperitoneal fibrosis or active inflammation, which would have been consistent with the diagnostic criteria for these diseases. Finally, we diagnosed EAF of the lung. The p-ANCA levels decreased postoperatively (12.1 U/mL), and the presenting cough had improved. We recommended regular follow-up, and the patient has so far been recurrence-free.

## Discussion

EAF is a rare disorder that involves the upper respiratory tract, which is diagnosed on the basis of the characteristic pathological findings, as previously mentioned [1–3]. The histopathological diagnosis takes two possible disease progression states into consideration: an inflammatory state and a fibrotic state [4, 5]. It is considered that the disease progresses from the early-state inflammatory lesion, with numerous eosinophils and perivascular onion skin fibrosing to a late state characterized by subsequent perivascular fibrosis formation and decreased inflammatory infiltration. In our case, the resected specimen contained these metachronous states, with an early state predominance. The enhanced accumulation on FDG-PET reflected the inflammatory cell infiltration and indicated a high-activity lesion. EAF of the lung should be considered in the differential diagnosis of a hilar mass lesion with abnormal accumulation that mimics lung cancer.

The etiology of EAF is unknown; atopy, trauma, surgery, and immunologic disease have been proposed as predisposing factors, but none of these is supported by evidence [6, 7]. Deshpande et al. reported five cases of EAF and concluded that EAF belongs to a spectrum of IgG4-related disorders [4]. In their cases, numerous IgG4-positive plasma cells have been observed in EAF biopsy samples, together with elevated serum IgG4 levels [8]. Although our case showed similar pathological findings, other laboratory results did not concur. We did not reach the definite the diagnosis for IgG4-related disease.

The differential diagnosis of pulmonary EAF includes GPA and eosinophilic GPA (EGPA) [3, 9], both systemic diseases with lung involvement. The most important histopathological distinguishing features are necrosis and vasculitis, which are not observed in EAF in our case. In addition, our patient lacked the diagnostic clinical findings associated with GPA and EGPA [10, 11]. The only positive serum assay was p-ANCA, which are the autoantibodies observed characteristically in EGPA. However, they are observed only in half of the EGPA cases, and the criteria proposed by the American College of Rheumatology do not include p-ANCA [11]. Our patient had not any clinically associated presentation. Consequently, we did not reach the definite diagnosis for these diseases.

While EAF is a benign disorder that involves the upper respiratory tract and leads to airway obstruction, the obstructive symptoms develop gradually because EAF is slow growing and progressive. Kim et al. reported a case of EAF that occurred in the bronchial lumen of a patient who presented with respiratory distress [5]. Similarly, in our case, chronic cough appeared as a local manifestation and disappeared after surgical treatment. If bronchial narrowing had progressed, disorders such as obstructive pneumonia would have developed. The medical treatment of EAF is not established. Therefore, surgical treatment for symptomatic relief was required in most of the reported cases. Whenever surgical resection is deemed feasible, it is desirable to intervene before the obstruction becomes severe. Among the cases where surgical excision was performed, one recurrence was described following a non-total resection [3]. Even in the case of a pulmonary lesion, a total resection may be required, and we therefore insist on the confirmation of the surgical margin by intraoperative rapid diagnosis.

In our case, EAF was not associated with a co-existing systemic disease. As previously described, EAF is an inflammatory disorder of uncertain origin. However, it is not known if EAF is a partial manifestation of a systemic illness, which highlights the importance of performing an adequate evaluation. As reported by Rimmer et al. [12], the possibility of advancing toward the development of these diseases is an important concern. Careful follow-up after surgery is also important.

## Conclusions

We report a surgical case of EAF of the lung that mimicked lung cancer. EAF is an unidentified benign disorder, but its growth can adversely affect organ function. In the case of a pulmonary lesion, it is desirable to completely remove it surgically before the development of airway obstruction. In addition, it is necessary to explore the possibility of an underlying systemic disease before making the diagnosis.

## Consent

Written informed consent was obtained from the patient for publication of this case report and any accompanying images. A copy of the written consent is available for review by the Editor-in-Chief of this journal.
